# Evaluation of isocitrate dehydrogenase mutation in 2021 world health organization classification grade 3 and 4 glioma adult-type diffuse gliomas with 18F-fluoromisonidazole PET

**DOI:** 10.1007/s11604-023-01450-x

**Published:** 2023-05-23

**Authors:** Yang Wang, Yasutaka Fushimi, Yoshiki Arakawa, Yoichi Shimizu, Kohei Sano, Akihiko Sakata, Satoshi Nakajima, Sachi Okuchi, Takuya Hinoda, Sonoko Oshima, Sayo Otani, Takayoshi Ishimori, Masahiro Tanji, Yohei Mineharu, Kazumichi Yoshida, Yuji Nakamoto

**Affiliations:** 1 Department of Diagnostic Imaging and Nuclear Medicine, Graduate School of Medicine, Kyoto University, 54 Shogoin Kawahara-Cho, Sakyo-Ku, Kyoto, 606-8507 Japan; 2 Department of Neurosurgery, Graduate School of Medicine, Kyoto University, Kyoto, 606-8507 Japan; 3 Division of Clinical Radiology Service, Kyoto University Hospital, Kyoto, 606-8507 Japan

**Keywords:** Hypoxia, FMISO, PET-CT, *IDH* mutation, Glioma

## Abstract

**Purpose:**

This study aimed to investigate the uptake characteristics of ^18^F-fluoromisonidazole (FMISO), in mutant-type *isocitrate dehydrogenase* (*IDH-mutant,* grade 3 and 4) and wild-type *IDH* (*IDH-wildtype*, grade 4) 2021 WHO classification adult-type diffuse gliomas.

**Materials and methods:**

Patients with grade 3 and 4 adult-type diffuse gliomas (n = 35) were included in this prospective study. After registering ^18^F-FMISO PET and MR images, standardized uptake value (SUV) and apparent diffusion coefficient (ADC) were evaluated in hyperintense areas on fluid-attenuated inversion recovery (FLAIR) imaging (HIA), and in contrast-enhanced tumors (CET) by manually placing 3D volumes of interest. Relative SUV_max_ (rSUV_max_) and SUV_mean_ (rSUV_mean_), 10th percentile of ADC (ADC_10pct_), mean ADC (ADC_mean_) were measured in HIA and CET, respectively.

**Results:**

rSUV_mean_ in HIA and rSUV_mean_ in CET were significantly higher in *IDH-wildtype* than in *IDH-mutant* (P = 0.0496 and 0.03, respectively). The combination of FMISO rSUV_mean_ in HIA and ADC_10pct_ in CET, that of rSUV_max_ and ADC_10pct_ in CET, that of rSUV_mean_ in HIA and ADC_mean_ in CET, were able to differentiate *IDH-mutant* from *IDH-wildtype* (AUC 0.80). When confined to astrocytic tumors except for oligodendroglioma, rSUV_max_, rSUV_mean_ in HIA and rSUV_mean_ in CET were higher for *IDH-wildtype* than for *IDH-mutant*, but not significantly (P = 0.23, 0.13 and 0.14, respectively). The combination of FMISO rSUV_mean_ in HIA and ADC_10pct_ in CET was able to differentiate *IDH-mutant* (AUC 0.81).

**Conclusion:**

PET using ^18^F-FMISO and ADC might provide a valuable tool for differentiating between *IDH* mutation status of 2021 WHO classification grade 3 and 4 adult-type diffuse gliomas.

**Supplementary Information:**

The online version contains supplementary material available at 10.1007/s11604-023-01450-x.

## Introduction

The radiotracer ^18^F-fluoromisonidazole (FMISO) accumulates in hypoxic viable cells after reduction reactions in the absence of oxygen. PET using ^18^F-FMISO allows the detection of hypoxia associated with the rapid depletion of nutrients that occurs with the abnormal proliferation of tumor cells seen in glioma [1, 2]. Hypoxia is associated with resistance to radiotherapy and chemotherapy in gliomas, and is related to the outcomes of glioma therapies [3]. Despite the importance of clarifying the extent of hypoxia in gliomas, common imaging modalities cannot clearly identify hypoxia in gliomas.

Isocitrate dehydrogenase (*IDH*) mutation is known to affect the prognosis of patients with glioma [4–6], and the knowledge of *IDH* mutation has been incorporated into 2021 WHO classification of brain tumors [7]. Prediction of *IDH* mutation by imaging would facilitate the optimization of therapeutic strategies for gliomas. Previous reports have demonstrated that 2-hydroxyglutarate (2-HG), which accumulates in *IDH*-mutated gliomas, can be detected by magnetic resonance spectroscopy [8]. On the other hand, PET probes have been reported to potentially allow prediction of *IDH* mutation status. Recent studies have found significant associations between ^18^F-fluoro-ethyl-tyrosine (^18^F-FET) PET results and *IDH* mutation status [9–11]. Another recent paper investigated the association of 3'-deoxy-3'-^18^F-fluorothymidine (^18^F-FLT) PET and ^18^F-FMISO PET, as well as relative cerebral blood volume in 31 patients with glioblastoma [12].

As ^18^F-FMISO PET is known to be useful in differentiating glioma grades [13], we hypothesized that there might be some association between *IDH* mutation status and ^18^F-FMISO uptake in glioma. The present study aimed to investigate the characteristics of ^18^F-FMISO uptake by 2021 WHO classification grade 3 and 4 glioma in terms of *IDH* mutation status.

## Materials and methods

### Patients

**Fig. 1:**
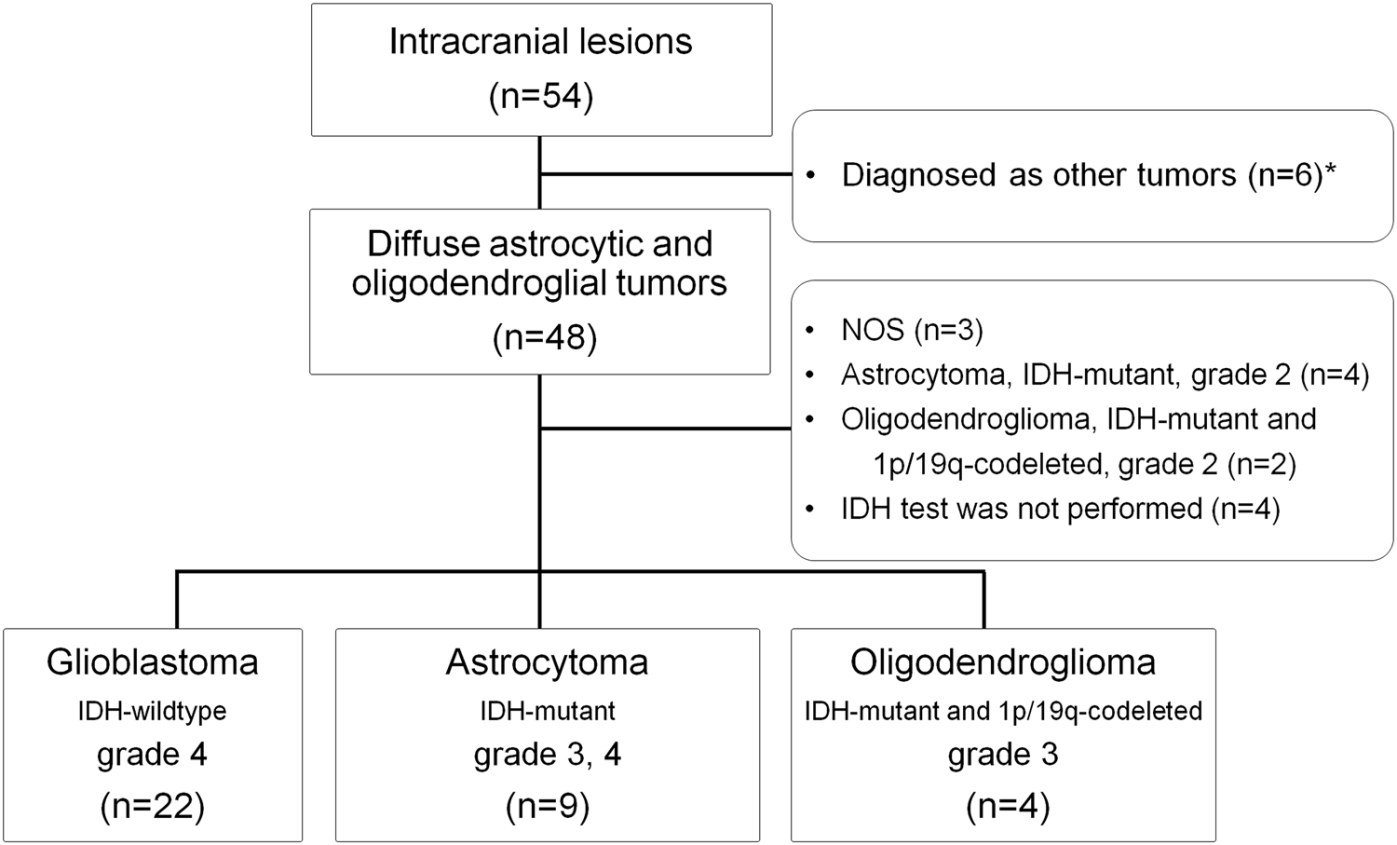
A flowchart of inclusion and exclusion criteria for this study. A total of 35 patients were included in this study (Glioblastoma, *IDH*-*wildtype*, n = 22; Astrocytoma, *IDH*-*mutant*, grade 3, 4, n = 9; Oligodendroglioma, *IDH-mutant*, and *1p/19q-codeleted*, grade 3, n = 4). Note that patients who were histopathologically diagnosed with other brain tumors were excluded from our study (n = 6) (*). Five of them were histopathologically diagnosed as schwannoma 2, metastatic tumor 1, central neurocytoma 1, ependymoma 1. One patient was clinically diagnosed as pilocytic astrocytoma without surgery at the time of PET scan, but was recently operated due to tumor volume increase, and histopathologically diagnosed as pilocytic astrocytoma

The institutional ethics committee approved this prospective study. Patients who were suspected intracranial brain lesions were enrolled in this study between September 2015 and March 2018, and written informed consent was obtained from each patient. In cases where the patient could not provide a signature, another family member provided informed consent instead. Tumors were included or excluded according to 2021 WHO classifications [7]. Figure 1 shows the inclusion and exclusion criteria. First, we included patients with histopathological diagnoses of 2021 WHO classifications grade 3 and 4 glioma (n = 35), which includes: (a) Glioblastoma, *IDH-wildtype*, grade 4 (n = 22); (b) Astrocytoma, *IDH-mutant*, grade 3, 4 (n = 9); (c) Oligodendroglioma, *IDH-mutant* and *1p/19q-codeleted*, grade 3 (n = 4). Second, patients who were histopathologically diagnosed with other brain tumors were excluded from our study (n = 6). Third, glioma, not otherwise specified (NOS) were excluded from our study (n = 3). Fourth, grade 2 gliomas were excluded from our study (Astrocytoma, *IDH-mutant*, n = 4; Oligodendroglioma, *IDH-mutant* and *1p/19q-codeleted*, n = 2). Fifth, *IDH* testing was not performed on four patients because histopathology was performed between 2011 and 2014 or because the specimens were not in suitably good condition, so those four patients were excluded from the study (n = 4). *IDH* testing was performed with an immunohistochemistry assay.

### PET protocol

Static PET images of the brain were acquired 4 h after intravenous injection of 350–550 MBq of ^18^F-FMISO. All images were acquired using a PET/CT scanner (Discovery IQ; GE Healthcare, Milwaukee, WI) with 5 circular BGO (Bi_4_Ge_3_O_12_) detectors. Low-dose CT was obtained as follows: 120 kVp; transaxial field of view (FOV), 500 × 500 mm; matrix size, 512 × 512; slice thickness, 3.75 mm. Next, emission scan was performed for 15 min and images were reconstructed with attenuation correction using CT data: transaxial FOV, 250 × 250 mm; matrix size, 192 × 192; axial FOV, 250 mm; voxel size, 1.3 × 1.3 × 3.3 mm; post-filtering at 2 mm full-width at half-maximum, VUE Point HD (3D ordered subsets expectation–maximization, OSEM), point spread function (subsets, 12; iterations, 4; filter cutoff, 2.0 mm).

### MR protocol

Brain MRI was conducted using a 3-T MRI unit (MAGNETOM Prisma or Skyra; Siemens Healthineers, Erlangen, Germany) with a 64-channel head/neck coil or a 32-channel head coil, including the following image sequences: non-enhanced (NE) and contrast-enhanced (CE) 3-dimensional (3D) T1-weighted imaging (T1WI): TR/TE, 6.0/2.3 ms; flip angle (FA), 15º; FOV, 230 × 230 mm^2^; resolution 0.9 × 0.9 mm^2^; slice thickness, 0.9 mm. Fluid-attenuated inversion recovery (FLAIR): TR/TE/TI, 12,000/100/2760 ms; FA, 120º; FOV, 220 × 192 mm^2^; resolution 0.69 × 0.69 mm^2^; slice thickness, 4 mm. Apparent diffusion coefficient (ADC) map was created from diffusion-weighted imaging (b = 0, 1000 s/mm^2^): TR/TE, 5000/77 ms; FA, 90º; FOV 220 × 220 mm^2^; resolution, 0.69 × 0.69 mm^2^; slice thickness, 4 mm. Gadolinium-based contrast agent (GBCA, 0.1 mmol/kg) was administered intravenously for CE-T1WI.

### Image processing and parameter calculation

#### Registration

Images from ^18^F-FMISO PET, FLAIR, ADC map, and CE 3D T1WI were registered to NE 3D T1WI images using SPM12 (https://www.fil.ion.ucl.ac.uk/spm). Images from NE 3D T1WI were segmented into gray matter, white matter, and other parts using SPM12.

#### Definition of volume of interest (VOI)

**Fig. 2:**
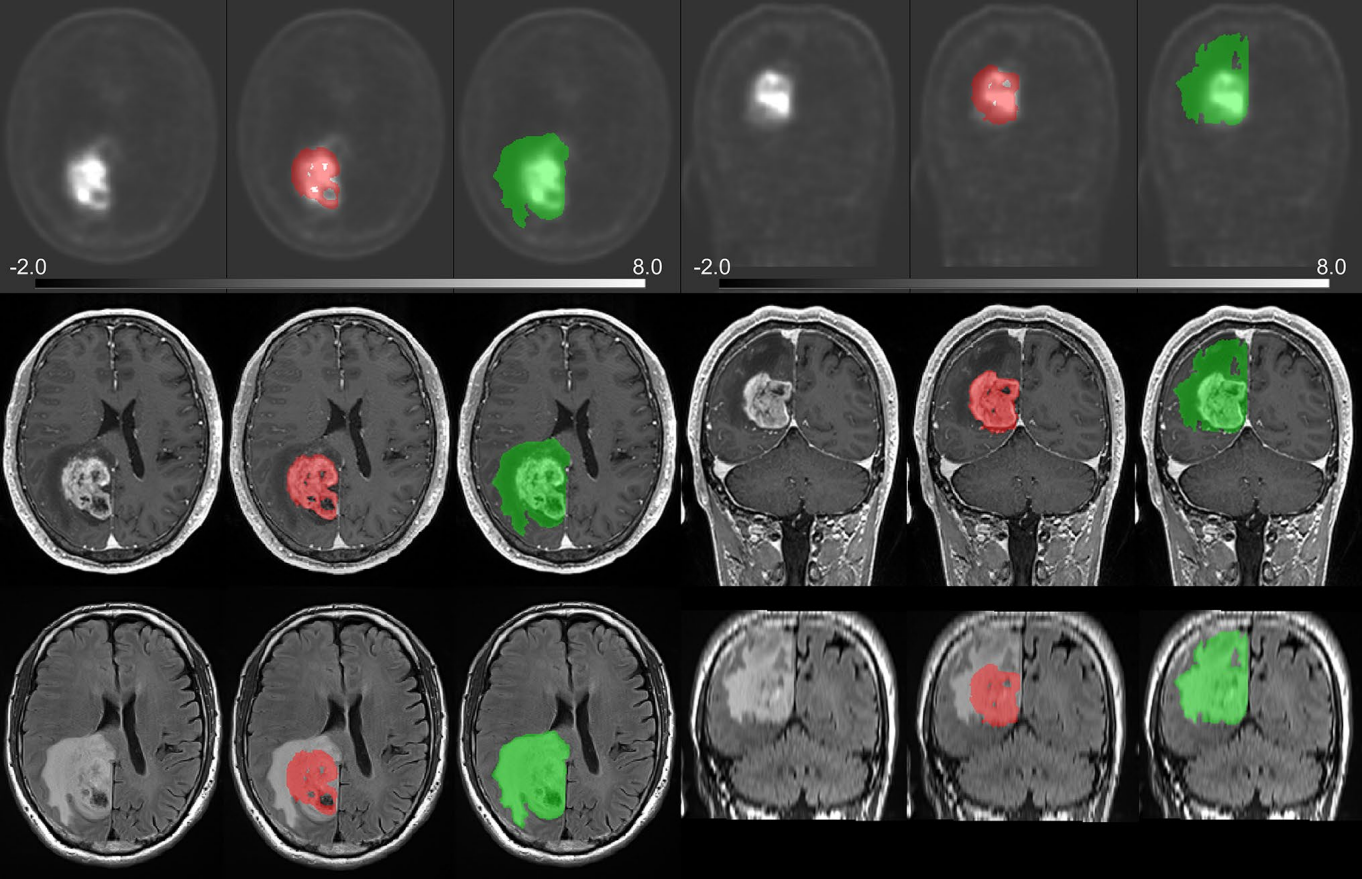
Representative VOIs for the case with Glioblastoma, *IDH*-wildtype are shown. FMISO (top), CE T1WI (middle row), FLAIR (bottom). Hyperintense areas on FLAIR imaging (HIA), representing areas of hyperintensity around and inside the tumor on FLAIR imaging (green), contrast-enhancing tumors (CET), representing tumor areas limited by the area of hyperintensity on CE T1WI (red)

Two VOIs were manually placed by a board-certified radiologist with 8 years of experience in neuroradiology, using ITK-SNAP software (https://www.itksnap.org) [14] and approved by another board-certified radiologist with 22 years of experience in neuroradiology. (a) Hyperintense areas on FLAIR imaging (HIA) were defined as areas of hyperintensity around and inside the tumor on FLAIR imaging. (b) Contrast-enhanced tumors (CET) were defined as areas of tumor enhancement on CE T1WI. Areas of central hypointensity on CE T1WI were considered to represent regions of central necrosis and were excluded from among VOIs of CET. Hyperintense areas on NE T1WI were considered to represent hemorrhagic lesions and were removed from among the VOIs of CET. Representative VOIs are shown in Fig. 2. Cerebellar cortical VOIs were created for reference using segmented cerebellar cortices.

### Data analysis

Relative standardized uptake value (SUV) of ^18^F-FMISO PET images was calculated as follows: SUV_max_, SUV_mean_ in HIA, and SUV_mean_ in CET were divided by the SUV_mean_ of cerebellar cortical VOIs, respectively. This resulted in rSUV_max_ and rSUV_mean_ in HIA, and rSUV_mean_ in CET.

We measured ADC_10pct_ in HIA, ADC_mean_ in HIA, ADC_10pct_ in CET, and ADC_mean_ in CET using the ADC map of MRI images [15, 16]. MANGO software (Research Imaging Institute, UTHSCSA, http://ric.uthscsa.edu/mango/) was used for these measurements.

We performed the following analyses to determine *IDH* mutation status of glioma.

#### IDH-mutant vs. IDH-wildtype

We compared rSUV_max_ and rSUV_mean_ in HIA, rSUV_mean_ in CET, ADC_10pct_ and ADC_mean_ in HIA, and ADC_10pct_ and ADC_mean_ in CET between *IDH-mutant* and *IDH-wildtype* in terms of following: A) comparisons between *IDH-mutant* and *IDH-wildtype* in all patients (n = 35); B) in patients in astrocytic tumors (n = 31).

### Statistical analysis

We applied the Mann–Whitney U test for measured values that did not follow a normal distribution.

We also performed logistic regression analysis with the above-mentioned rSUV and ADC, followed by receiver operating characteristic (ROC) curve analysis. Areas under the curve (AUCs) were calculated with optimal cutoff, sensitivity, and specificity in terms of following: (A) determination of *IDH* mutation status in all patients; (B) determination of *IDH* mutation status in patients in astrocytic gliomas; AUCs were compared with DeLong test.

All statistical analyses were performed using JMP version 15 software (SAS Institute, Cary, NC, United States). Values of P < 0.05 were considered significant.

## Results

### Patients

**Fig. 3:**
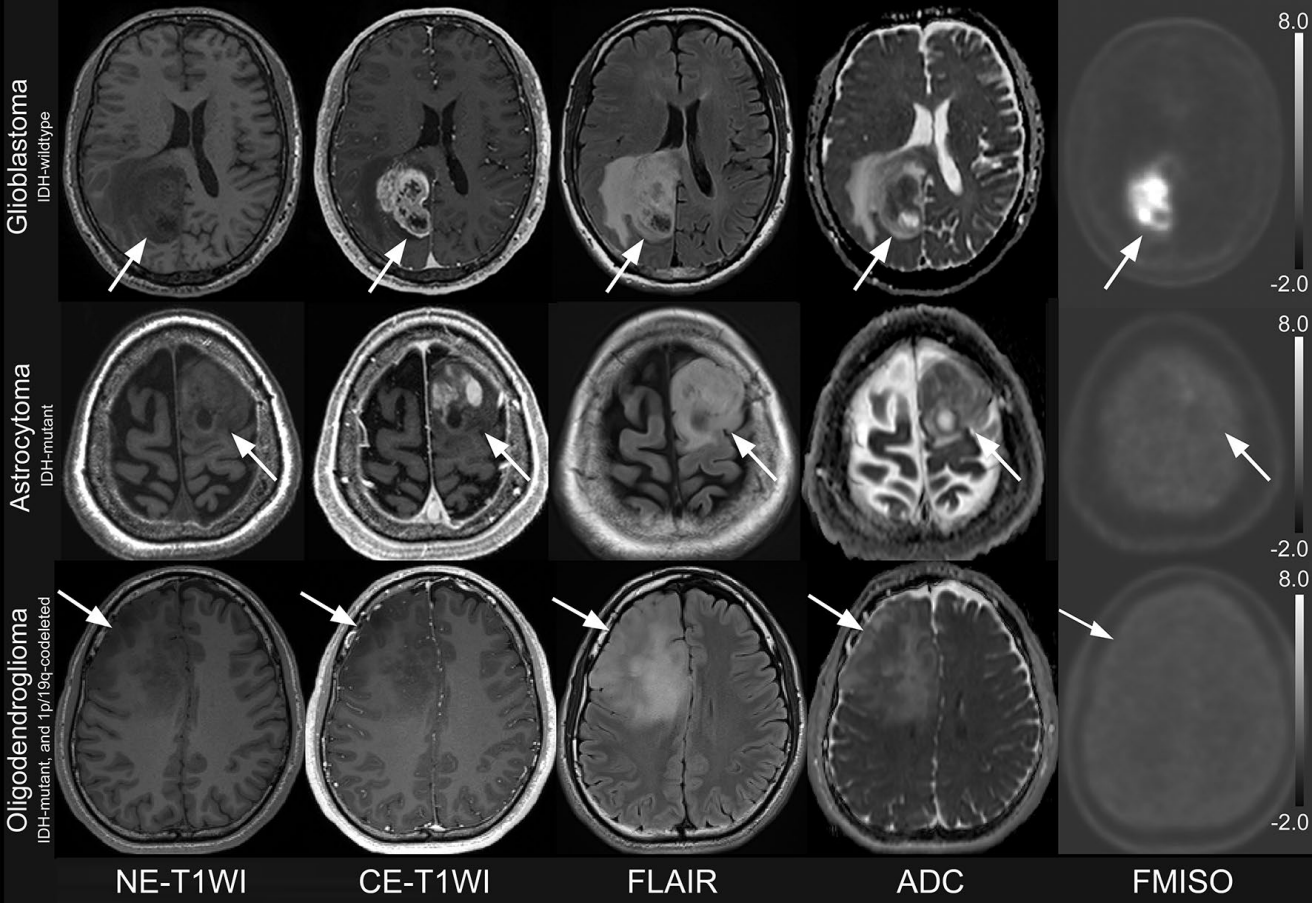
Three representative cases are shown. A 60-year-old man with Glioblastoma, *IDH-wildtype*, grade 4 (top); A 67-year-old woman with Astrocytoma, *IDH*-*mutant*, grade 4 (second row); A 24-year-old man with Oligodendroglioma, *IDH-mutant*, and *1p/19q-codeleted*, grade 3 (bottom). FMISO-PET shows prominent uptake in glioblastoma, *IDH-wildtype*, and moderate uptake in Astrocytoma, *IDH-mutant*, grade 4. Meanwhile, FMISO-PET shows low uptake in Oligodendroglioma, *IDH-mutant*, and *1p/19q-codeleted*

A total of 35 patients were included in this study. The demographic characteristics of patients are shown in Table 1 and Fig. 1. rSUV and ADC in *IDH-mutant* and *IDH-wildtype* are shown in Table 2. No registration error was observed. Representative images are shown in Fig. 3.

**Table 1:** Demographic characteristics of patients

Patients	
Age [years]	50 [38–64]
Sex	20 males, 15 females
IDH1 mutation	
Mutant	13
Wildtype	22
Pathological diagnosis	
Astrocytoma, IDH-mutant, grade 3	2
Astrocytoma, IDH-mutant, grade 4	7
Oligodendroglioma, IDH-mutant, and 1p/19q-codeleted, grade 3	4
Glioblastoma, IDH-wildtype, grade 4	22

**Table 2:** Results of SUV, ADC with and without *IDH* mutation

	All (n = 35)		Astrocytic tumor (n = 31)	
	IDH-wildtype (n = 22)	IDH-mutant (n = 13)	IDH-wildtype (n = 22)	IDH-mutant (n = 9)
rSUV_max_	3.15 [1.63–4.26]	2.11 ± 0.72	3.15 [1.63–4.26]	2.35 ± 0.72
rSUV_mean_ in HIA	1.17 [1.01–1.42]	1.05 ± 0.10	1.17 [1.01–1.42]	1.06 ± 0.11
rSUV_mean_ in CET	1.63 [1.23–2.04]	1.09 [1.06–1.52]	1.63 [1.23–2.04]	1.20 [1.07–1.68]
ADC_10pct_ in HIA [10^−3^mm^2^/sec]	0.78 ± 0.09	0.83 ± 0.11	0.78 ± 0.09	0.81 ± 0.10
ADC_10pct_ in CET [10^−3^mm^2^/sec]	0.83 ± 0.15	0.83 ± 0.22	0.83 ± 0.15	0.80 ± 0.18
ADC_mean_ in HIA [10^−3^mm^2^/sec]	1.07 [0.99–1.21]	1.17 ± 0.12	1.07 [0.99–1.21]	1.17 ± 0.11
ADC_mean_ in CET [10^−3^mm^2^/sec]	1.08 [0.97–1.29]	1.16 ± 0.19	1.08 [0.97–1.29]	1.16 ± 0.19

Data are mean ± standard deviation, and when the data did not show normal distribution, data are median [interquartile range]. rSUV_mean_ in CET in *IDH-mutant*; rSUV_max_, rSUV_mean_ in HIA, rSUV_mean_ in CET, ADC_mean_ in HIA and ADC_mean_ in CET in *IDH-wildtype*

#### Comparisons between IDH-mutant and IDH-wildtype in all patients (n = 35)

**Fig. 4:**
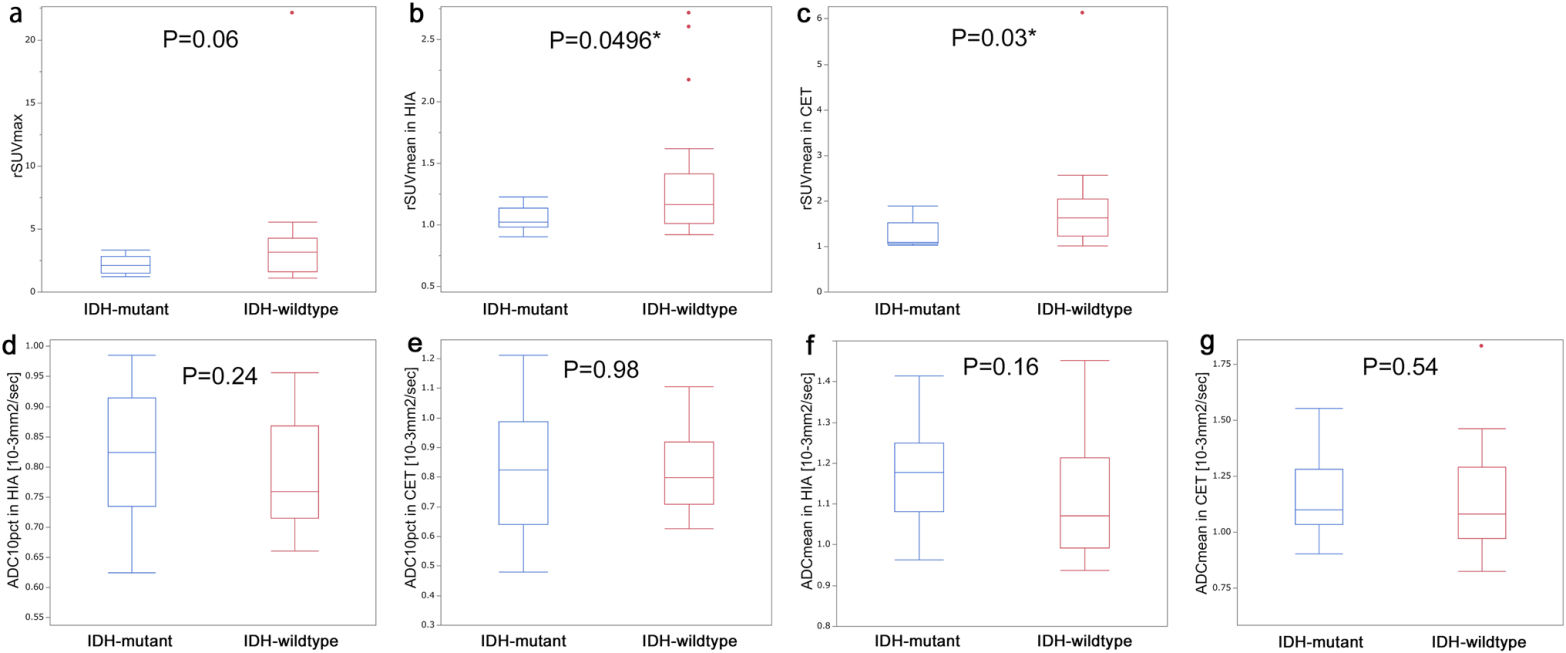
Comparison between *IDH-mutant* (n = 13) and *IDH*-*wildtype* (n = 22) in all patients. The rSUV_mean_ in HIA (**b**), and rSUV_mean_ in CET (**c**) are significantly higher for *IDH*-*wildtype* than for *IDH*-*mutant*. The rSUV_max_ (**a**) tend to be higher for *IDH*-*wildtype* than for *IDH*-*mutant*, but not significantly. The ADC_10pct_ in HIA (**d**) and ADC_mean_ in HIA (**f**) tend to be lower for *IDH*-*wildtype* than for *IDH*-*mutant*, but not significantly. No significant differences were found in ADC_10pct_ in CET (**e**) and ADC_mean_ in CET (**g**). Asterisks (*) represent statistically significant differences

rSUV_mean_ in HIA and rSUV_mean_ in CET were significantly higher for *IDH-wildtype* than for *IDH-mutant* (P = 0.0496 and 0.03, respectively) (Fig. 4). rSUV_max_ were higher for *IDH-wildtype* than for *IDH-mutant* but not significantly (P = 0.06).

ADC_10pct_ in HIA and ADC_mean_ in HIA were lower for *IDH-wildtype* than for *IDH-mutant* but not significantly (P = 0.24 and 0.16, respectively). No significant differences were found in ADC_10pct_ in CET or ADC_mean_ in CET (P = 0.98 and 0.54, respectively).

#### Comparisons between IDH-mutant and IDH-wildtype in patients in astrocytic tumors (n = 31)

**Fig. 5:**
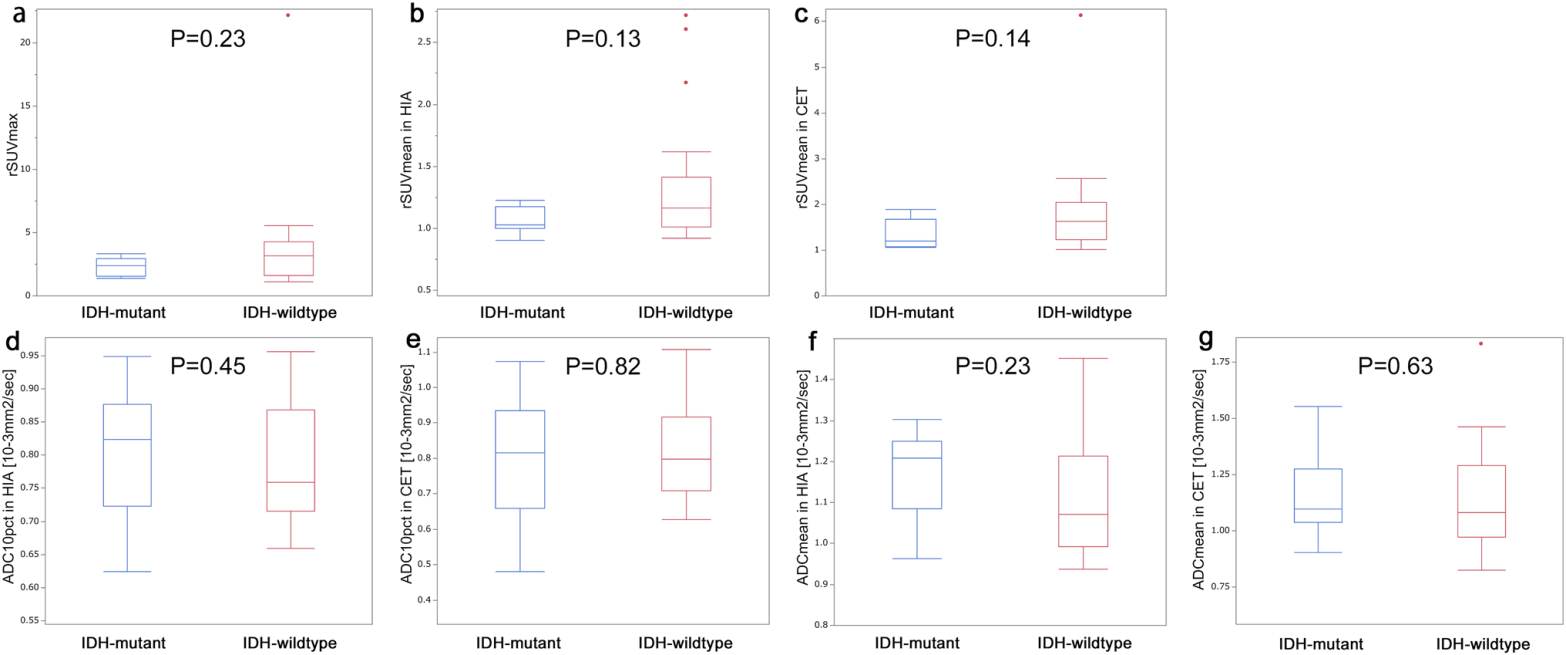
Comparison between *IDH-mutant* (n = 9) and *IDH-wildtype* (n = 22) in astrocytic tumors. The rSUV_max_ (**a**), rSUV_mean_ in HIA (**b**) and rSUV_mean_ in CET (**c**) were higher for *IDH-wildtype* than for *IDH-mutant*, but not significantly. The ADC_10pct_ in HIA (**d**) and ADC_mean_ in HIA (**f**) were lower for *IDH-wildtype* than for *IDH-mutant* but not significantly. No significant differences were found in ADC_10pct_ in CET (**e**) or ADC_mean_ in CET (**g**)

rSUV_max_, rSUV_mean_ in HIA and rSUV_mean_ in CET were higher for *IDH-wildtype* than for *IDH-mutant*, but not significantly (P = 0.23, 0.13 and 0.14, respectively) (Fig. 5).

ADC_10pct_ in HIA and ADC_mean_ in HIA were lower for *IDH-wildtype* than for *IDH-mutant* but not significantly (P = 0.45 and 0.23, respectively). No significant differences were found in ADC_10pct_ in CET or ADC_mean_ in CET (P = 0.82 and 0.63, respectively).

### ROC curve analysis

**Fig. 6:**
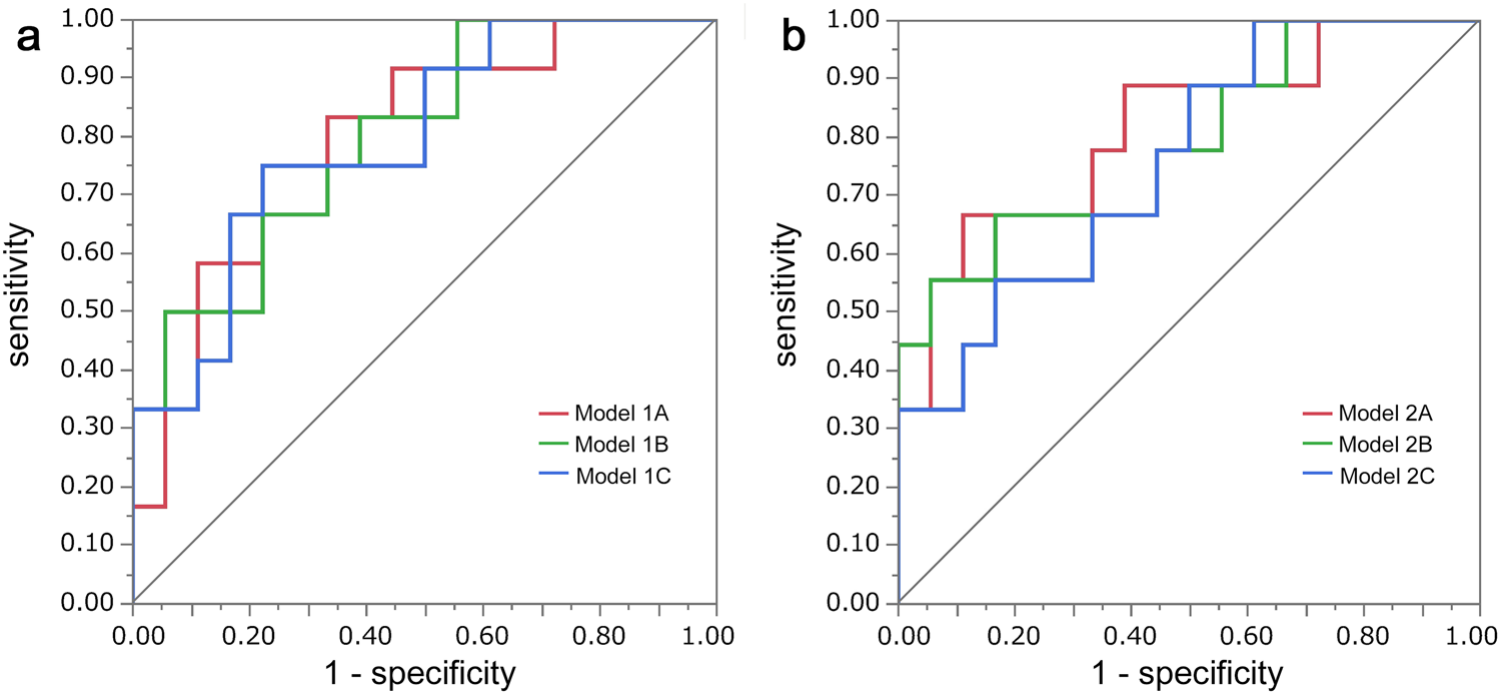
ROC analysis was performed for 2 groups. The highest three AUCs were shown for each group. **a** Prediction of *IDH* mutation status in all patients (n = 35): Model 1A, rSUV_mean_ in HIA and ADC_10pct_ in CET (AUC, 0.80); Model 1B, rSUV_max_ and ADC_10pct_ in CET (AUC, 0.80); Model 1C, rSUV_mean_ in HIA and ADC_mean_ in CET (AUC, 0.80). **b** Prediction of *IDH* mutation status in the patients of astrocytic tumors (n = 31): Model 2A, rSUV_mean_ in HIA and ADC_10pct_ in CET (AUC, 0.81); Model 2B, rSUV_max_ and ADC_10pct_ in CET (AUC, 0.79); Model 2C, rSUV_mean_ in HIA and ADC_mean_ in CET (AUC, 0.76)

ROC curve analysis was performed using logistic regression analysis with rSUV and ADC to determine glioma *IDH* mutations status in all patients (n = 35) (Fig. 6a) and in astrocytic tumors (n = 31) (Fig. 6b). AUCs of all parameters are shown in Supplemental Tables 1 and 2.

The best three models for *IDH* mutation status in all patients (n = 35) were Model 1A, 1B and 1C. Model 1A, rSUV_mean_ in HIA and ADC_10pct_ in CET (AUC, 0.80). The optimal cutoffs (rSUV_mean_ in HIA 1.02; ADC_10pct_ in CET 1.07) offered 75.0% sensitivity and 77.8% specificity; Model 1B using rSUV_max_ and ADC_10pct_ in CET (AUC, 0.80). The optimal cutoffs (rSUV_max_ 2.19; ADC_10pct_ in CET 1.07) offered 83.3% sensitivity and 61.1% specificity; Model 1C, rSUV_mean_ HIA and ADC_mean_ in CET (AUC, 0.80). The optimal cutoffs rSUV_mean_ HIA 1.02; ADC_mean_ in CET 1.55) offered 75.0% sensitivity and 77.8% specificity (Fig. 6a). DeLong test showed no statistical significance in AUC was observed between each model (Supplemental Table 3).

The best model for *IDH* mutation status in astrocytic tumor patients (n = 31) was Model 2A, using rSUV_mean_ in HIA and ADC_10pct_ in CET (AUC, 0.81). The optimal cutoffs (rSUV_mean_ in HIA 1.05; ADC_10pct_ in CET 0.82) offered 66.7% sensitivity and 88.9% specificity; Model 2B using rSUV_max_ and ADC_10pct_ in CET (AUC, 0.79). The optimal cutoffs (rSUV_max_ 2.81; ADC_10pct_ in CET 0.62) offered 55.6% sensitivity and 94.4% specificity; Model 2C, rSUV_mean_ HIA and ADC_mean_ in CET (AUC, 0.76). The optimal cutoffs rSUV_mean_ HIA 1.05; ADC_mean_ in CET 1.28) offered 55.6% sensitivity and 83.3% specificity (Fig. 6b). DeLong test showed no statistical significance in AUC was observed between each model (Supplemental Table 3).

## Discussion

We were able to differentiate *IDH* mutation status using rSUV_mean_ in HIA and ADC_10pct_ in CET, with AUC of 0.80 in all patients, and AUC of 0.81 in the patients with astrocytic tumors, according to 2021 WHO classification. Obviously, FMISO does not directly reflect *IDH* mutation status. A recent study revealed that multidrug-resistant protein 1 (MRP1) inhibitors increase ^18^F-FMISO accumulation in hypoxic cells. This suggests that ^18^F-FMISO PET imaging is affected by MRP1 inhibitors independent of the state of hypoxia [17]. FMISO uptake is known to reflect hypoxic circumstances in tissues, and increased tumor aggressiveness induces greater hypoxia inside the tumor [18–21]. *IDH* mutation is considered to offer a strong predictor of less-aggressive glioma [22–24], and FMISO would thus indirectly reflect *IDH* mutation status.

In the present study, no significant difference in ADC was apparent between *IDH-wildtype* and *IDH-mutant*. However, diagnostic performance increased after combining ADC with SUV. Our study demonstrated that rSUV_mean_ in HIA and ADC_10pct_ in CET can differentiate *IDH* mutation status with high diagnostic ability in grade 3, 4 gliomas by 2021 WHO classification (AUC = 0.80, in all patients; AUC = 0.81, in astrocytic tumors, respectively). In the literature, detection of *IDH* mutation status was possible in grade 2 and 3 glioma of 2016 WHO classification, using the ratio of ADC_mean_ to ADC of normal-appearing white matter (AUC 0.83) [25], ADC ratio (AUC 0.95) [26], minimum ADC (ADC_min_) (AUC 0.87), and relative ADC_min_ (AUC 0.84) [27]. Meanwhile, in grade 3, 4 gliomas, *IDH* mutation status was able to be differentiated by ADC_mean_ (AUC 0.71), ADC_10pct_ (AUC 0.71) from histogram study [28], and ratio of ADC_min_ to normal white matter (AUC = 0.70) in grade 4 glioma [29]. Previous study also showed that, FMISO tumor–blood SUV ratio (TBR) could differentiate *IDH*-*mutant* type from *IDH*- *wildtype* in grade 3, 4 gliomas (AUC = 0.78 in all patients, AUC = 0.76 in astrocytic tumors) [30]. Our results were comparable to the those of previous studies.

*IDH* mutation status has some association with other PET tracers. The ratios of the SUV_max_ of the tumors to the SUV_mean_ of the contralateral cortex (T/N ratios) of FLT-PET/CT can be used to determine the *IDH* mutation status with an AUC of 0.911; The T/N ratios of ^11^C-methionine (MET) methionine can be used to determine the *IDH* mutation status with an AUC of 0.727 [31]. Based on 2016 WHO classification, the differences in mean ^18^F-FLT tumor-normal tissue ratio (TNR) and ^18^F-FMISO TBR were significant between GBM and other glioma subtypes (P < 0.001); and regarding the comparison between Gd-T1WI volumes and ^18^F-FLT MTVs or ^18^F-FMISO MTVs, previous study identified significant differences between *IDH*-*wildtype* and *IDH-mutant* or *1p19q-*codeletion (P < 0.01) [32]. The percentage difference between the standard biological tumor volume (BTV) on standard summation images and BTV on early summation images could differentiate *IDH* mutation status with an AUC of 0.83 using ^18^F-FET PET [10]. Time-to-peak value in a dynamic ^18^F-FET PET study also showed good diagnostic performance for *IDH* mutation status in gliomas (AUC 0.75) [33]. While 3D-VOIs were manually created for HIA and CET in our study, as in a previous study [34], 2-dimensional regions of interest (2D-ROI) were used in most studies examining ^18^F-FMISO PET. Our study registered images from PET and MRI, and 3D-segmented cerebellar cortices were also used as references to calculate relative SUV, although 2D-ROIs were used as reference for ^18^F-FMISO uptakes in previous articles [18, 34, 35]. 3D-VOIs for HIA may underestimate rSUV_mean_ of ^18^F-FMISO uptake because 3D-VOIs for HIA are larger than those for CET. However, rSUV_max_ can compensate such underestimation in HIA, since physiological ^18^F-FMISO uptake is low and homogeneous in the brain parenchyma compared with other amino acid PET tracers.

Some limitations need to be acknowledged in this study. First, the number of patients enrolled was small (n = 35). More patients need to be enrolled to confirm the present results. Second, biodistribution of ^18^F-FMISO has not been evaluated in this study. ^18^F-FMISO is relative lipophilic and diffuses through cell membranes, and mild uptake of ^18^F-FMISO is seen in normal tissue [36]. In addition, the previous study had performed dynamic ^18^F-FMISO and dynamic ^15^O-H_2_O PET in brain tumors to measure tumor hypoxia and perfusion, and increased ^18^F-FMISO tumor retention at late scan time was found predominantly in glioblastoma, but not found in meningiomas, which lacks the blood brain barrier (BBB) [35]. Their data suggested that late ^18^F-FMISO PET images obtained 4 h after the injection provide a spatial description of hypoxia in brain tumors that is independent of BBB disruption and tumor perfusion. Third, dynamic susceptibility contrast perfusion weighted imaging (DSC-PWI), which is beneficial in differentiation *IDH-wildtype* glioma and *IDH-mutant* glioma, was not used in this study. *IDH* mutation status of grade 2 and 3 gliomas of 2016 WHO classification can be differentiated by relative CBV_max_ with an AUC of 0.82 [27]. *IDH* mutation status in glioblastoma could be differentiated by rCBV_mean_ with AUC of 0.886 [37]. *IDH* mutation status could be differentiated using Visually AcceSAble Rembrandt Images (VASARI) MRI feature set in grade 2 and 3 gliomas, grade 2 glioma only, and grade 3 glioma only with AUCs of 0.78, 0.83 and 0.87, respectively [38].

## Conclusions

In conclusion, PET using ^18^F-FMISO and ADC might provide a valuable tool for differentiating *IDH* mutation status of 2021 WHO classification grade 3 and 4 adult-type diffuse glioma.

### Supplementary Information

Below is the link to the electronic supplementary material.

Supplementary file1 (DOCX 44 KB)

Supplementary file2 (DOCX 17 KB)

Supplementary file3 (DOCX 17 KB)
