# Supplementary material for: Evaluation of isocitrate dehydrogenase mutation in 2021 world health organization classification grade 3 and 4 glioma adult-type diffuse gliomas with 18F-fluoromisonidazole PET
Source: Jpn J Radiol. 2023 May 23;41(11):1255–64. doi: 10.1007/s11604-023-01450-x (PMC10613590; doi:10.1007/s11604-023-01450-x)
Supplement: Supplementary file 1 — Supplementary file1 (DOCX 44 KB) [file 11604_2023_1450_MOESM1_ESM.docx]

**Supplemental Table 1**

ROC analysis was performed for (a) differentiation of *IDH* mutation status in all patients (n = 35), (b) differentiation of *IDH* mutation status in astrocytic tumor patients (n = 31) with models including CET. Models with the highest three AUCs are shown in Fig. 4. AUCs for the same values were ranked in order including the third decimal place. *IDH-mutant*, mutant-type isocitrate dehydrogenase gene; *IDH-wildtype*, wild-type isocitrate dehydrogenase gene; ADC, apparent diffusion coefficient; HIA, hyperintense areas on FLAIR imaging; CET, contrast-enhancing tumors

(a) Differentiation of *IDH* mutation status in all 35 patients

| Model | AUC [95% Cl] |
| --- | --- |
| rSUV_max_ and ADC_10pct_ in HIA | 0.70 [0.51, 0.85] |
| rSUV_max_ and ADC_10pct_ in CET | 0.80 [0.60, 0.92] |
| rSUV_max_ and ADC_mean_ in HIA | 0.72 [0.52, 0.86] |
| rSUV_max_ and ADC_mean_ in CET | 0.76 [0.55, 0.89] |
| rSUV_mean_ in HIA and ADC_10pct_ in HIA | 0.72 [0.53, 0.86] |
| rSUV_mean_ in HIA and ADC_10pct_ in CET | 0.80 [0.59, 0.92] |
| rSUV_mean_ in HIA and ADC_mean_ in HIA | 0.72 [0.52, 0.86] |
| rSUV_mean_in HIA and ADC_mean_ in CET | 0.80 [0.59, 0.91] |
| rSUV_mean_ in CET and ADC_10pct_ in HIA | 0.75 [0.54, 0.89] |
| rSUV_mean_ in CET and ADC_10pct_ in CET | 0.75 [0.54, 0.89] |
| rSUV_mean_ in CET and ADC_mean_ in HIA | 0.75 [0.53, 0.88] |
| rSUV_mean_ in CET and ADC_mean_ in CET | 0.74 [0.51, 0.88] |
| rSUV_max_ | 0.70 [0.50, 0.84] |
| rSUV_mean_ in HIA | 0.70 [0.51, 0.85] |
| rSUV_mean_ in CET | 0.74 [0.52, 0.88] |
| ADC_10pct_ in HIA | 0.62 [0.40, 0.81] |
| ADC_10pct_ in CET | 0.64 [0.28, 0.72] |
| ADC_mean_ in HIA | 0.65 [0.44, 0.81] |
| ADC_mean_ in CET | 0.57 [0.36, 0.76] |

(b) Differentiation of *IDH* mutation status in astrocytic tumor patients (n = 31)

| Model | AUC [95% Cl] |
| --- | --- |
| rSUV_max_ and ADC_10pct_ in HIA | 0.64 [0.44, 0.80] |
| rSUV_max_ and ADC_10pct_ in CET | 0.79 [0.54, 0.92] |
| rSUV_max_ and ADC_mean_ in HIA | 0.70 [0.50, 0.85] |
| rSUV_max_ and ADC_mean_ in CET | 0.71 [0.48, 0.87] |
| rSUV_mean_ in HIA and ADC_10pct_ in HIA | 0.69 [0.47, 0.85] |
| rSUV_mean_ in HIA and ADC_10pct_ in CET | 0.81 [0.57, 0.94] |
| rSUV_mean_ in HIA and ADC_mean_ in HIA | 0.69 [0.48, 0.84] |
| rSUV_mean_in HIA and ADC_mean_ in CET | 0.76 [0.52, 0.90] |
| rSUV_mean_ in CET and ADC_10pct_ in HIA | 0.70 [0.48, 0.86] |
| rSUV_mean_ in CET and ADC_10pct_ in CET | 0.73 [0.48, 0.89] |
| rSUV_mean_ in CET and ADC_mean_ in HIA | 0.67 [0.44, 0.84] |
| rSUV_mean_ in CET and ADC_mean_ in CET | 0.67 [0.42, 0.85] |
| rSUV_max_ | 0.64 [0.44, 0.81] |
| rSUV_mean_ in HIA | 0.68 [0.46, 0.84] |
| rSUV_mean_ in CET | 0. 68 [0.44, 0.85] |
| ADC_10pct_ in HIA | 0.59 [0.34, 0.80] |
| ADC_10pct_ in CET | 0.53 [0.29, 0.76] |
| ADC_mean_ in HIA | 0.64 [0.40, 0.83] |
| ADC_mean_ in CET | 0.56 [0.34, 0.76] |
