# Supplementary material for: Evaluation of isocitrate dehydrogenase mutation in 2021 world health organization classification grade 3 and 4 glioma adult-type diffuse gliomas with 18F-fluoromisonidazole PET
Source: Jpn J Radiol. 2023 May 23;41(11):1255–64. doi: 10.1007/s11604-023-01450-x (PMC10613590; doi:10.1007/s11604-023-01450-x)
Supplement: Supplementary file 2 — Supplementary file2 (DOCX 17 KB) [file 11604_2023_1450_MOESM2_ESM.docx]

**Supplemental Table 2**

The volume of HIA and CET.

Volume of HIA and CET

|  | All (n = 35) | Astrocytic tumors (n = 31) |
| --- | --- | --- |
| HIA [ml] | 84.2 ± 58.6 | 85.4 ± 60.2 |
| CET [ml] | 12.3 [3.56, 39.7] | 12.3 [4.26, 38.5] |

Data are mean ± standard deviation, and when the data did not show normal distribution, data are median [interquartile range].

AUC of HIA and CET

|  | All (n = 35) | Astrocytic tumors (n = 31) |
| --- | --- | --- |
| HIA | 0.57 | 0.59 |
| CET | 0.59 | 0.62 |

HIA, hyperintense areas on FLAIR imaging; CET, contrast-enhanced tumor.
