# Supplementary material for: Evaluation of isocitrate dehydrogenase mutation in 2021 world health organization classification grade 3 and 4 glioma adult-type diffuse gliomas with 18F-fluoromisonidazole PET
Source: Jpn J Radiol. 2023 May 23;41(11):1255–64. doi: 10.1007/s11604-023-01450-x (PMC10613590; doi:10.1007/s11604-023-01450-x)
Supplement: Supplementary file 3 — Supplementary file3 (DOCX 17 KB) [file 11604_2023_1450_MOESM3_ESM.docx]

**Supplemental Table 3**

Models of AUCs for differentiation of IDH mutation status were compared by using DeLong

1. Results of DeLong test (all patients, n = 35)

| Models | rSUV_mean_ in HIA and ADC_10pct_ in CET | rSUV_mean_ in CET | ADC _mean_ in HIA |
| --- | --- | --- | --- |
| rSUV_mean_ in HIA and ADC_10pct_ in CET | - | - | - |
| rSUV_mean_ in CET | 0.48 | - | - |
| ADC _mean_ in HIA | 0.23 | 0.46 | - |

1. Results of DeLong test (astrocytic tumor patients, n = 31)

| Models | rSUV_mean_ in HIA and ADC_10pct_ in CET | rSUV_mean_ in CET | ADC _mean_ in HIA |
| --- | --- | --- | --- |
| rSUV_mean_ in HIA and ADC_10pct_ in CET | - | - | - |
| rSUV_mean_ in CET | 0.25 | - | - |
| ADC _mean_ in HIA | 0.27 | 0.79 | - |
